# Leisure Time Physical Activity and Associated Factors among Adults in Estonia 2000–2018

**DOI:** 10.3390/ijerph18063132

**Published:** 2021-03-18

**Authors:** Marii Mikk, Inge Ringmets, Kersti Pärna

**Affiliations:** Institute of Family Medicine and Public Health, University of Tartu, Ravila 19, 50411 Tartu, Estonia; marii.mikk@gmail.com (M.M.); inge.ringmets@ut.ee (I.R.)

**Keywords:** leisure time physical activity, adults, socioeconomic factors, body mass index, self-rated health, smoking, alcohol use, Estonia

## Abstract

In order to implement evidence-based strategies, there is a need to assess (1) time trend in leisure time physical activity (LTPA) and (2) the relationship between trend of LTPA and trend of potential explanatory factors in Estonia from 2000 to 2018. Data from 25−64-year-old adults (*n* = 16,903) were drawn from cross-sectional surveys of Health Behavior among Estonian Adult Population. Joinpoint regression analysis was used to calculate annual percentage changes (APCs) and to identify whether there was a significant change in trends of LTPA. Logistic regression analysis was used to assess associations of LTPA with socioeconomic, health-related and health-behavioral factors. Prevalence of LTPA increased from 26.2% to 44.1% among men and from 28.0% to 40.6% among women from 2000 to 2018 (*p* < 0.001). Average APC for men was 3.4% (95% CI 2.6−4.3) and for women 2.4% (95% CI 1.4−3.4). Adjusted logistic regression model showed that LTPA was statistically significantly associated with higher education and income, economic inactivity, at-least-good self-rated health (SRH) and non-smoking. Interaction of SRH with study year was significant indicating that the association of at-least-good SRH changed over time (*p* = 0.016). Health promotion activities should be addressed in particular to adults with lower levels of LTPA, paying attention to the factors associated with LTPA.

## 1. Introduction

The importance and benefits of physical activity (PA) have been widely studied [1,2,3]. The recommended PA level by the World Health Organization (WHO) for healthy adults aged 18−64 years is at least 150 min of moderate-intensity activity or 75 min of vigorous-intensity activity per week, or an equivalent combination of activities of both intensities. The duration of PA should be at least 10 min, with a minimum frequency of 3 times a week. In addition, it is recommended to perform muscle-strengthening activities affecting major muscle groups at least twice a week [4,5].

PA is roughly divided into leisure-time, occupational and transportation related PA [6]. Leisure-time physical activity (LTPA), indicating activities engaged in outside working hours [7], is the most prevalent physical activity in the modern societies. Over the years, LTPA has increased in several countries. In Finland, high LTPA has increased in men from 21% to 33% and women from 12% to 27% in 1982–2012 [8]. In Poland, LTPA increased among men and women in 2014–2018 reaching to 43.9% and 43.5%, respectively [9]. In Norway, LTPA decreased from 23.2% to 16.0% in 1979–2001 and subsequently increased to 24.3% in 2008 [10].

According to the worldwide literature, LTPA is associated with different factors [11,12,13,14]. The results of Eurobarometer survey demonstrated that men were more likely to be physically active than women in 2017, especially in younger age groups (15 to 24 year olds) [11]. At the same time, a cross-sectional study in Spain showed that among seniors (≥65 years old), women were physically more active than men [12]. Many studies have investigated the associations between LTPA and socioeconomic status (SES), specifically through the mediators of education and income [13]. In a systematic review, in which most of the included studies were conducted in Scandinavian countries, adults with lower SES engaged in less vigorous LTPA than those with higher SES [14].

In the global literature was found that LTPA can be associated with body mass index (BMI) in different ways. For example, higher BMI was seen as a result of lower LTPA [15] as well as the reason for higher LTPA [16]. Additionally, a sufficient level of LTPA affects health-related self-perception. Kaleta et al. [17] found that men who expend at least 1000 kcal a week during leisure time had significantly lower odds of rating their health as poor compared to men who were not physically active during leisure time. The same study suggested that women who were physically inactive during leisure time had significantly higher odds of rating their health as poor compared to women who were physically active during leisure time.

Recent studies have found that risk behaviors are correlated with LTPA. Not being a smoker was associated with higher LTPA [10,18]. Moreover, daily smoking increased also the likelihood of remaining or becoming physically inactive over the decades [19]. Findings concerning association between LTPA and alcohol consumption are not entirely consistent. Some studies confirmed, that LTPA was negatively correlated with alcohol consumption [18], several studies did not find any association between PA and alcohol consumption [20], and some studies found positive association between LTPA and alcohol consumption [21].

To implement evidence-based strategies to increase LTPA, it is necessary to identify the trends in LTPA and associated factors in a country. The aim of this study was to describe the trend in LTPA and to analyze the relationship between trend of LTPA and trend of socioeconomic, health-related and health-behavioral factors among adults in Estonia in 2000–2018.

## 2. Materials and Methods

### 2.1. Dataset and Sample

The present study was based on secondary data from postal cross-sectional surveys of Health Behavior among Estonian Adult Population, which have been conducted biennially since 1990 using a harmonized methodology and questionnaire. A stratified random sampling of the Estonian population aged 16–64 years was based on population registry residences. Until 2002, the initial sample consisted of 2000 individuals, but since 2004, 5000 individuals have been included. The highest corrected response rate (excluding those who did not live at the address provided, those for whom no letter box was available, those not living in Estonia, and those who had died) was 77.4% in 2000, and the lowest was 51.4% in 2018 [22,23].

All surveys were approved by the Tallinn Medical Research Ethics Committee in Estonia. Along with the questionnaires, the recipients received a separate survey information sheet. It provided detailed description of the survey methodology including its ethical aspects (autonomy, data handling, etc.). Survey participation was, therefore, considered as informed consent which is quite common practice for large-scale population surveys [22,23].

The present study included adults aged 25–64 years from ten surveys of Health Behavior among Estonian Adult Population from 2000 to 2018 (*n* = 18,916). Age restriction was used to minimize the effects of the potential misclassification of the dependent variable and SES among younger respondents.

### 2.2. Variables

#### 2.2.1. Main Outcome Variable

The main outcome variable was LTPA based on the question: “How often in your leisure time do you exercise for at least half an hour so that you breathe slightly heavier and sweat a little?”. In line with WHO recommendations, the answer options were divided into two groups: physically active (every day, 4–6 times a week, 2–3 times a week) and physically inactive (once a week, 2–3 times a month, a few times a year/not at all).

#### 2.2.2. Explanatory Variables

All explanatory variables were dichotomized to simplify the interpretation of the results. However, dichotomizing could lead to model underestimation and, therefore, the results should be interpreted as minimum estimates.

The socioeconomic factors were as follows: education, income and employment status. Education was based on the highest completed educational level and was classified into “1 = higher education (15+ years)”, “0 = secondary (10–14 years) or basic (less than 10 years) education”. Income was determined by the average monthly income per family member. Data were categorized into “1 = high (higher than median)” and “0 = low (lower than median)”. Employment status was categorized into “1 = economic activity (working)”, “0 = economic inactivity (unemployed or not working or retired)”.

Health-related factors were BMI and self-rated health (SRH). BMI was categorized as “1 = overweight (25+ kg/m^2^)” and “0 = normal weight or less (<25 kg/m^2^)”. SRH was categorized as “1 = at-least-good” and “0 = less-than-good”.

Health-behavioral factors were smoking status and alcohol consumption. Smoking status was categorized as “1 = current smoking” and “0 = non-smoking”. Alcohol consumption was categorized as “1 = drinking at once six units of alcohol (one unit = 10 g of pure alcohol) at least once a week” and “0 = less often or never”.

#### 2.2.3. Control Variables

Control variables were gender (men; women), age (categorical variable), and ethnicity (Estonian; non-Estonian).

### 2.3. Statistical Analysis

Joinpoint regression analysis was used to calculate annual percentage changes (APCs) in LTPA and to identify whether there was a significant (*p* < 0.05) change in trend of LTPA among men and women. As the LTPA among men and women was similar, the further analysis was performed for men and women together. Chi-square-test for trend was used to describe changes in explanatory factors over the study period. Logistic regression analysis was used to test associations between LTPA (yes vs. no) and socioeconomic, health-related and health-behavioral factors. Logistic regression model was fitted for the outcome variable while adjusting for control variables (age, gender, ethnicity). The model included interaction terms between time (categorical variable) and each explanatory variable. Odds ratios (ORs) with 95% confidence intervals (CIs) for each year were calculated from the full model as marginal estimates. Chi-square goodness-of-fit test was used to assess model fit.

Questionnaires where the answer to the LTPA question was missing (*n* = 538) or when the answer was that the individual’s health status does not allow physical activity due to injury or illness (*n* = 1954), were excluded from the analysis. In total, 18,916 adults (40.4% men and 59.6% women) aged 25–64 from 2000 to 2018 were included in the data analysis (Table 1).

The data were analyzed using the statistical program Stata 14.2 (StataCorp, College Station, TX, USA) [24].

## 3. Results

### 3.1. Trend in LTPA and Explanatory Factors

In 2000–2018, proportion of adults with higher LTPA, education and income, with economic activity, overweight, at-least-good SRH and alcohol use increased, but smoking decreased significantly (Table 2).

**Figure 1:**
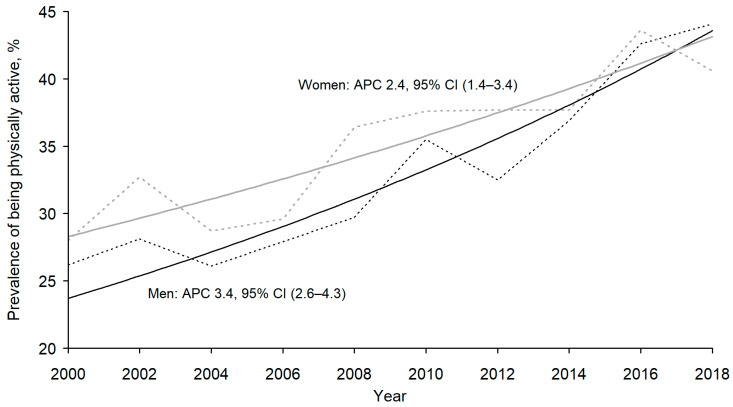
Trends in the prevalence of leisure time physical activity (LTPA) and respective annual percentage change (APC) by gender in Estonia 2000–2018. Dashed line marks the observed prevalence of LTPA, continuous line refers to annual percentage change for trend among men and women.

In 2000, 26.2% of men and 28.0% of women and in 2018, 44.1% and 40.6%, respectively, were physically active during the leisure time. The prevalence of LTPA among men and women increased significantly (*p* < 0.001) from 2000 to 2018 (Figure 1). Joinpoint regression analysis showed that for men, the average annual percent change during the study period was 3.4% (95% CI 2.6 to 4.3) and for women 2.4% (95% CI 1.4 to 3.4). In 2000–2016, the prevalence of LTPA was somewhat lower among men than women, but in 2018 this was higher among women.

### 3.2. Association between LTPA and Explanatory Factors over the Study Period

The adjusted odds ratios for each study years showed that LTPA was associated with education, income, employment status, SRH and smoking status (Table 3). The LTPA was higher among adults with higher education since 2004. Significant slight association between LTPA and higher income was found in 2008, 2014 and 2018 only. The LTPA was significantly associated with economic inactivity over study period except in 2012 and 2018. However, the changes in association between LTPA and socioeconomic factors over time were not significant. The association between LTPA and at-least-good health was statistically significant over the study period. Interaction of SRH with study year was also significant indicating that the relationship between LTPA and at-least-good SRH changed in time (*p* = 0.016 for interaction). The closer look by study year revealed that the association between LTPA and at-least-good SRH decreased sharply during the first 3 study years and started to increase again since 2012. The association between LTPA and being non-smoker was statistically significant since 2002. Although this association seems to become somewhat weaker over the study period, the changes were not statistically significant in time. The LTPA was not associated with BMI and alcohol use. Goodness-of-fit test showed that the model is correctly specified (*p* = 0.099).

## 4. Discussion

The present study focused on the trend of LTPA and the relationship between LTPA and socioeconomic, health-related and health-behavioral factors among adults in Estonia in 2000–2018.

### 4.1. Trend in LTPA

This study revealed that LTPA increased significantly from 2000 to 2018 in Estonia. The findings of the previous studies in European countries are in line with this study [8,9,10,12,25,26]. The underlying reasons for increased levels of LTPA in Estonia can only be speculated, but it is possible that the systematic promotion of physical activity (creation of prerequisits and opportunities for health development, raising people’s awareness of these opportunities and health behavior) over the last decades as one activities of National Health Plan in 2009−2020 may have had a role in this change [27]. Moreover, the coverage of light traffic routes and the number of sports clubs have increased over the last years in Estonia, which provides more opportunities for people to be physically active. Urbanization may also have some effect on the growth in LTPA because this allows people better and faster access to different sports facilities.

In 2000–2016, the LTPA was slightly higher among women, but in 2018 somewhat higher among men in Estonia. Whether this will remain the case, will be clear in coming years. Eurobarometer survey (2017) [11] and studies in most European countries have reported higher LTPA among men or have identified gender equity in sports participation [28].

### 4.2. Association between LTPA and Explanatory Factors over the Study Period

The adjusted logistic regression model showed that LTPA among adults was statistically significantly associated with education, income, employment status, SRH and smoking status in Estonia. No association was found between LTPA and BMI as well as between LTPA and alcohol consumption.

The LTPA was associated with higher SES among adults in Estonia and there was not found significant changes in these associations in 2000–2018 in Estonia. Previous systematic review has reported that in most developed countries those with high SES were more physically active during leisure-time compared to those with low SES [14]. While in this study the association between LTPA and high income was found only in some study years, then the relationship between LTPA and higher education existed over the study period and association between LTPA and economic inactivity over the first half of the study period. Adults with higher levels of education tend to be more aware of health risks, so in this case, the relationship between LTPA and higher education was expected. Moreover, education level is available for both men and women, including those who are currently outside employment. At the same time, it is worth mentioning that LTPA does not necessarily involve additional costs because one can also be physically active just by moving in nature. Higher LTPA among economically inactive adults might be explained by the fact, that people who work have less time for LTPA [29]. On the other hand, LTPA would have been expected to be higher among economically active adults since the present study found an association between LTPA and higher income. It can be assumed that more engagement in LTPA among economically inactive people was enabled by the income of a family member. At the same time, it could also be explained by the possibility that economically inactive people used more cost-efficient ways to exercise, such as being physically active outdoors.

From health-related factors at-least-good SRH was strongly associated with LTPA over the study years. This association was expected because higher self-esteem provides favorable opportunities for LTPA and people, who are physically active, therefore, rate their health most likely as good. Additionally, this result is in line with previous studies [17]. In addition, the results of the present study indicated that the association between LTPA and at-least-good health changed significantly in time. This association decreased in the beginning of the study period, but started to increase during the last study years. One can speculate that adults with less-than-good SRH had to become more physically active in leisure time in 2004–2012.

Quite surprisingly, no association was found between LTPA and BMI in the current study. One reason for this could be, that self-reported height and weight used to calculate BMI may be not accurate. At the same time previous studies have shown association between LTPA and BMI [15,30,31]. It can be assumed that people who are overweight have a greater incentive to be physically active, while adults with obesity may find exercising physically difficult to perform or might lack the knowledge or confidence necessary to start engaging in LTPA. Additionally, it has been shown that overweight and obese people overreport physical activity [32], and also that they are seeing body weight as a barrier to being physically active [33].

Regarding health-behavioral factors, the association between LTPA and being non-smoker has remained strong and unchanged over the study period in Estonia. This finding is in accordance with previous literature [10,18,19].

No association was found between LTPA and alcohol consumption in this study. In the global literature was found that LTPA can be associated with alcohol consumption in different ways [18,20,21]. Several systematic reviews have reported association between slightly higher alcohol consumption and higher LTPA [34,35]. In addition, it is important to consider that different methods are often used in studies to describe alcohol consumption (amount vs. frequency) [34]. Using only alcohol consumption frequency questions may lead to erroneous results because, for example, a glass of wine on weekends with dinner cannot be equated with excessive alcohol consumption once a week.

### 4.3. Limitations and Strengths of the Survey

This survey has several limitations that need to be considered. *First*, the study relies on self-reported data. It is possible, that respondent’s LTPA, other health behaviors, height and weight could be affected by reporting bias [36]. Although these factors cannot be fully controlled for in the data, the social desirability bias is generally lower in self-administered postal surveys [37]. In addition, the LTPA question was used to determine the frequency of PA but did not take into account the intensity or total duration of PA since the survey did not solely focus on PA. *Second*, the overall response rates have been declining throughout the study years. Late response and item nonresponse in the Finbalt Health Monitor survey (including data from Estonia) has been analyzed earlier [38] and by assuming that non-respondents were similar to late respondents, the authors concluded that the response bias could be minimal. *Third*, this study is based on cross-sectional data, that does not allow establishing causality in associations.

The strengths of survey of Health Behavior among Estonian Adult Population are the similar study design and methodology across the years, which gives a great opportunity to analyze LTPA over decades in Estonia.

## 5. Conclusions

The findings of this study provide an evidence-based overview of increase in the prevalence of LTPA during last decades among adults in Estonia. LTPA was associated with higher SES, at-least-good SRH and being non-smoker over the study period. The relationships with SES and smoking status remained unchanged in time, but the association between LTPA and at-least-good SRH decreased significantly over the study years.

The findings of this study highlight the need for implementation of effective intervention strategies to increase LTPA that particularly target physically inactive adults with socioeconomic, health-related and health-behavioral predictors.

## Figures and Tables

**Table 1:** Study sample of 25–64-year-old men and women by study year in Estonia, 2000–2018.

Study Year	Men		Women		Total
	*n*	%	*n*	%	*n*
2000	397	41.2	567	58.8	964
2002	356	39.6	544	60.4	900
2004	915	2.2	1256	57.8	2171
2006	788	37.9	1289	62.1	2077
2008	925	42.2	1268	57.8	2193
2010	897	40.2	1334	59.8	2231
2012	881	40.1	1314	59.9	2195
2014	792	39.9	1191	60.1	1983
2016	883	40.3	1306	59.7	2189
2018	812	40.3	1201	59.7	2013
Total	7646	40.4	11,270	59.6	18,916

**Table 2:** The prevalence (%) of outcome and explanatory variables among adults in Estonia, 2000–2018.

Characteristic	2000	2002	2004	2006	2008	2010	2012	2014	2016	2018	*p*-Value
LTPA	27.3	30.9	27.6	29.0	33.6	36.8	35.6	37.4	43.2	42.0	<0.001
Higher education	21.3	22.1	21.7	26.3	29.1	30.1	32.2	34.1	37.2	43.9	<0.001
High income	42.3	45.1	47.5	47.3	46.9	43.1	49.7	46.9	49.6	47.1	0.008
Economic activity	77.0	78.2	78.6	81.5	83.9	74.8	78.2	79.2	80.3	83.9	0.005
BMI (25+ kg/m^2^)	46.8	48.0	49.1	51.7	55.8	54.2	53.0	54.3	54.8	53.4	<0.001
Good health	33.9	38.3	40.7	44.6	48.5	47.0	52.0	52.5	54.0	51.8	<0.001
Smoking	40.2	34.9	40.8	33.5	34.2	32.7	32.8	28.1	27.9	23.2	<0.001
Alcohol use	9.9	9.1	13.6	10.7	11.33	11.9	11.9	14.6	14.2	14.6	<0.001

**Table 3:** Association between LTPA (yes vs. no) and explanatory factors, 2000–2018, OR with 95% CI ^a^.

Variables	2000	2002	2004	2006	2008	2010	2012	2014	2016	2018	Interaction Year × Var *p*-Value
	OR (95% CI)	OR (95% CI)	OR (95% CI)	OR (95% CI)	OR (95% CI)	OR (95% CI)	OR (95% CI)	OR (95% CI)	OR (95% CI)	OR (95% CI)	
Higher education	1.13 (0.77−1.67)	0.89 (0.61−1.30)	1.46 (1.14−1.88)	1.26 (0.99−1.59)	1.41 (1.14−1.74)	1.63 (1.33−2.01)	1.52 (1.23−1.87)	1.57 (1.27−1.95)	1.33 (1.09−1.62)	1.47 (1.20−1.80)	0.209
High income	1.10 (0.78−1.54)	1.01 (0.73−1.40)	1.08 (0.86−1.35)	1.23 (0.99−1.52)	1.30 (1.07−1.59)	1.10 (0.90−1.34)	1.06 (0.87−1.30)	1.27 (1.03−1.57)	1.18 (0.97−1.43)	1.23 (1.01−1.51)	0.843
Economic activity	0.63 (0.43−0.93)	0.46 (0.32−0.67)	0.74 (0.57−0.97)	0.65 (0.50−0.85)	0.60 (0.46−0.78)	0.60 (0.48−0.75)	0.87 (0.69−1.11)	0.77 (0.61−0.99)	0.61 (0.48−0.77)	0.76 (0.58−1.00)	0.130
Overweight	1.02 (0.74−1.42)	1.00 (0.73−1.36)	0.93 (0.76−1.15)	0.98 (0.80−1.21)	0.99 (0.81−1.20)	1.10 (0.91−1.32)	1.04 (0.86−1.26)	1.02 (0.83−1.24)	0.97 (0.81−1.17)	0.91 (0.75−1.11)	0.976
Good health	2.26 (1.61−3.15)	1.67 (1.21−2.30)	1.20 (0.96−1.50)	1.52 (1.23−1.87)	1.52 (1.24−1.85)	1.37 (1.13−1.65)	1.30 (1.07−1.59)	1.54 (1.25−1.89)	1.27 (1.05−1.53)	1.85 (1.52−2.26)	0.016
Smoking	0.79 (0.56−1.11)	0.63 (0.45−0.88)	0.65 (0.52−0.82)	0.69 (0.55−0.87)	0.63 (0.51−0.78)	0.76 (0.62−0.93)	0.53 (0.42−0.66)	0.67 (0.53−0.85)	0.60 (0.48−0.74)	0.58 (0.46−0.74)	0.477
Alcohol use	0.76 (0.42−1.36)	1.21 (0.71−2.06)	1.10 (0.80−1.50)	1.14 (0.81−1.50)	0.81 0.59−1.13)	1.05 (0.78−1.40)	1.17 (0.86−1.60)	0.92 (0.69−1.24)	1.21 (0.92−1.59)	1.20 (0.90−1.59)	0.629

^a^ The model was adjusted for gender, age and ethnicity. All explanatory variables were included in the model at the same time with interaction terms with study year (year as categorical variable), *p*-values for the interaction were obtained from the full model. OR—odds ratio, 95% CI—95% confidence interval.

## Data Availability

The datasets analyzed in this study are available from coordinator of the survey Health Behavior among Estonian Adult Population on reasonable request.
